# Female reproductive success and calf survival in a North Sea coastal bottlenose dolphin (*Tursiops truncatus*) population

**DOI:** 10.1371/journal.pone.0185000

**Published:** 2017-09-20

**Authors:** Kevin P. Robinson, Texa M. C. Sim, Ross M. Culloch, Thomas S. Bean, Isabel Cordoba Aguilar, Sonja M. Eisfeld, Miranda Filan, Gary N. Haskins, Genevieve Williams, Graham J. Pierce

**Affiliations:** 1 Cetacean Research & Rescue Unit (CRRU), Banff, Scotland, United Kingdom; 2 Oceanlab, University of Aberdeen, Newburgh, Scotland, United Kingdom; 3 CESAM and Departamento de Biologia, Universidade de Aveiro, Aveiro, Portugal; 4 Instituto de Investigacións Mariñas (CSIC), Vigo, Spain; University of Missouri Columbia, UNITED STATES

## Abstract

Between-female variation in reproductive output provides a strong measure of individual fitness and a quantifiable measure of the health of a population which may be highly informative to management. In the present study, we examined reproductive traits in female bottlenose dolphins from the east coast of Scotland using longitudinal sightings data collected over twenty years. From a total of 102 females identified between 1997 and 2016, 74 mothers produced a collective total of 193 calves. Females gave birth from 6 to 13 years of age with a mean age of 8. Calves were produced during all study months, May to October inclusive, but showed a seasonal birth pulse corresponding to the regional peak in summer water temperatures. Approximately 83% (*n* = 116) of the calves of established fate were successfully raised to year 2–3. Of the known mortalities, ~45% were first-born calves. Calf survival rates were also lower in multiparous females who had previously lost calves. A mean inter-birth interval (IBI) of 3.80 years (*n* = 110) and mean fecundity of 0.16 was estimated for the population. Calf loss resulted in shortened IBIs, whilst longer IBIs were observed in females assumed to be approaching reproductive senescence. Maternal age and size, breeding experience, dominance, individual associations, group size and other social factors, were all concluded to influence reproductive success (RS) in this population. Some females are likely more important than others for the future viability of the population. Consequently, a better knowledge of the demographic groups containing those females showing higher reproductive success would be highly desirable for conservation efforts aimed at their protection.

## Introduction

In long-lived mammals, such as dolphins, that produce just one infant at a time, age at first birth and the spacing between births are the primary determinants for fecundity [1]. Female reproductive success (RS) is subsequently determined by the product of a reproductive life-span, the pace of parturition, and the survival rate of offspring (e.g. [2]). Thus, between-female variation in reproductive output provides a strong measure of individual fitness and a quantifiable measure of population health [3,4] which may be highly informative to conservation and management.

The Moray Firth (MF) in northeast Scotland (57°41ʹ N, 02°20ʹ W) contains the only year-round, resident population of common bottlenose dolphins (*Tursiops truncatus*) in inshore Scottish waters and the wider North Sea. Studies in this region have been conducted since the late 1980s and have greatly enhanced our understanding of this population and assisted in its management [5]. However, animals from the MF range widely beyond the designated Special Area of Conservation (SAC) established for their protection in the inner Moray Firth in 2005 [5–10], making robust estimations of individual birth rates and calf survival difficult to resolve for this population. Integrated datasets from multiple research sites have been used to provide a best estimate of ~200 animals using the northeast coastline [11]. Both males and females are seen to range widely along the east coast [12], but females are found to be more site-faithful within the SAC and adjacent outer MF regions [7,13]. Indeed, the southern coastline of the outer MF is thought to provide important summer calving / nursery areas for the population [6,7,14], in addition to other key areas utilised within the SAC [15]. During the summer and fall, large numbers of nursing females with calves use this region, with ~90% of the known population having been recaptured over the course of a 20-year mark-recapture study [Robinson unpublished data, 14].

Using longitudinal sightings data of known mothers and their calves, it has been possible to build a detailed description of the long-term reproductive histories of females from this population, from which individual variation in calf production, inter-birth intervals (IBIs) and the survival rates of calves could be investigated. In the following study, we explore the effects of maternal age, breeding experience, mother identity and birth month upon reproductive success and calf survival, and discuss the implication of these results for the future conservation of this potentially vulnerable North Sea bottlenose dolphin population.

## Methods

### Survey methods and photo-identification

**Fig 1:**
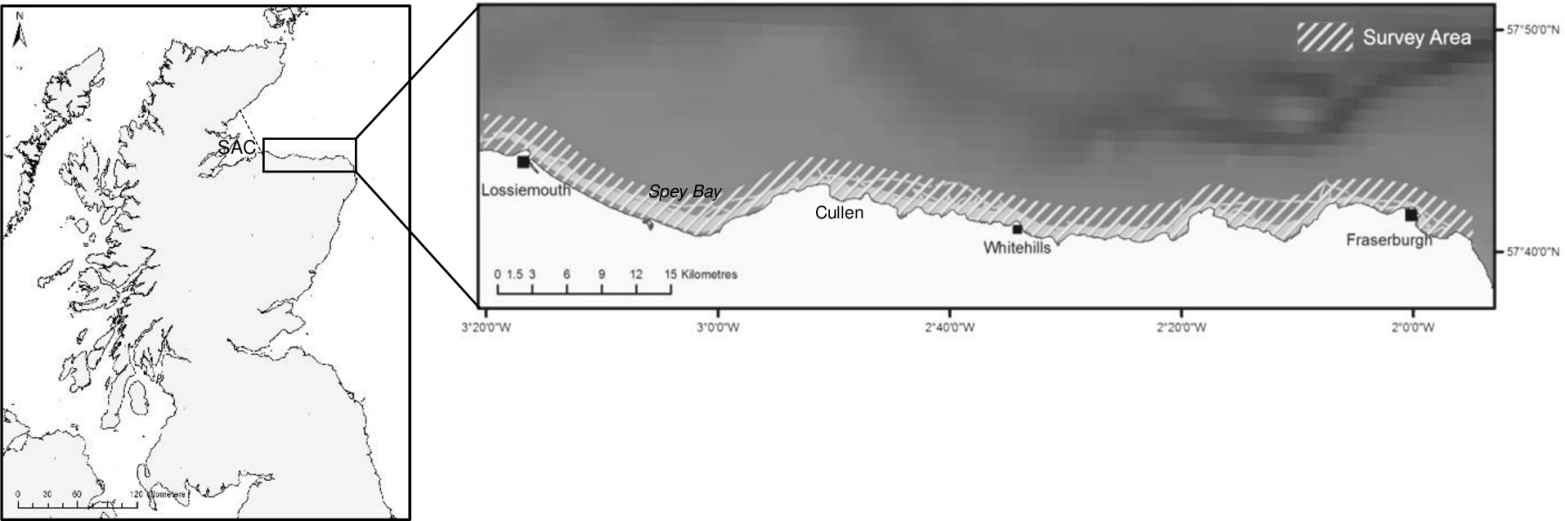
Map of northeast Scotland showing the position of the survey area along the southern coastline of the outer Moray Firth, to the east of the Special Area of Conservation (SAC). Dedicated boat surveys for bottlenose dolphins were conducted between May and October 1997 to 2016 using selected routes within the core study area, between the coastal ports of Lossiemouth and Fraserburgh.

Mark-recapture data were collected during dedicated boat surveys in the outer southern MF between May and October 1997 to 2016 inclusive. From 1997 to 2000, dedicated survey trips were carried-out using a variety of vessels, with effort primarily concentrated in the west of the study area between Spey Bay and Cullen (Fig 1). From 2001 onwards, boat surveys were conducted between the coastal ports of Lossiemouth and Fraserburgh using rigid inflatable boats, with selected routes chosen to maximise capture probabilities, whilst minimising sampling heterogeneity, within the core areas used by the dolphins. Survey effort was recorded from 2001 onwards and was variable between years. Consequently, to ascertain whether a relationship occurred between survey effort and the annual number of individuals, reproductive females or newborn calves recorded, a Pearson’s product-moment correlation was used.

Standardised photo-identification procedures were employed to obtain high-quality photographs of encountered animals using an SLR camera equipped with a f2.8 300 mm fixed lens. Transparency film was used from 1997 to 2006, after which digital imagery was used. All images were maintained within a purpose-designed photo-identification database (using Microsoft Access), from which a detailed record of the long-term sightings and calving histories for all reproductively-active females identified in the study area could be extracted. All boat-based research was carried-out under licence from Scottish Natural Heritage.

### Identification of mothers and calves

**Fig 2:**
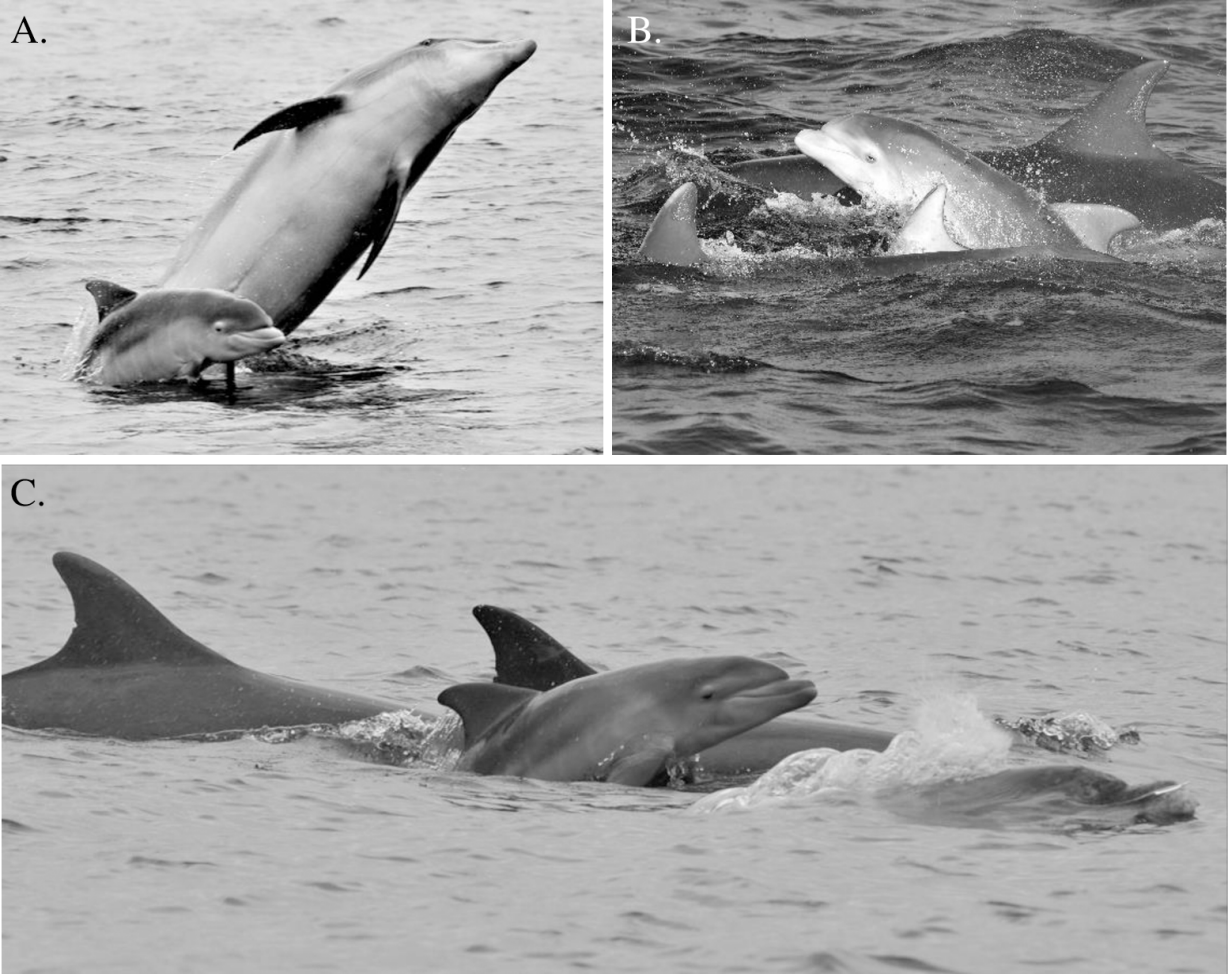
Photographs of female bottlenose dolphins from the Moray Firth with first-year calves: (A) one of the highly successful mothers (ID#065) with a new-born calf; (B) and (C) cooperative female groups with calves of similar ages in tow.

All mothers were identified from repeated recaptures with dependent calves in tow (Fig 2). In the absence of markings for photo-identification, calves were typically tracked from close association with their mothers until weaning. Nevertheless, temporary marks on the dorsal fin and body (e.g. lesions or scars/scratches, typically acquired with age [16]) also assisted in the short-term recapture of maturing infants. Furthermore, after maternal separation, from 2 to 4 years of age, former calves often remained philopatric, allowing their continued recapture by association through to maturity.

### Inter-birth intervals (IBIs) and reproductive success (RS)

The long-term nature of this study allowed retrieval of demographic data for the large majority of known females from this population, from which IBIs were determined for those producing two or more calves between 1997 and 2016 inclusive. Two approaches were used for IBI determination. The first approach examined all known IBIs (including those with large gaps between the weaning of one calf and the birth of the next), which potentially biased IBIs upwards due to failure to detect some births. The second approach removed females with IBIs >5 years, unless mothers remained in close association with the surviving calf until verification of a subsequent birth; potentially resulting in a downward bias in IBIs. IBIs for females that had experienced a calf loss, and IBIs between the first and the second born calves of primiparous females (of known birth year themselves) were compared to all known IBIs using unpaired t-tests.

A female was assumed to have reproduced successfully if her calf survived from birth (year 0–1) to the minimum age at weaning (year 1–2) [17]. Using this criterion, and to reduce the influence of small sample size on the estimated reproductive output, RS was only estimated for those females with three or more documented births. For the small subset of females of known age (i.e., individuals known since birth), the potential effects of the mother’s age on IBI's and RS were also examined qualitatively (there were insufficient data for quantitative analysis).

To determine the relationship between the likelihood of a calf being born and the preceding IBI, a binomial generalised additive mixed model (GAMM) was fitted, with calf birth (0 or 1) as the response variable, and IBI (in months) as the explanatory variable. Previous RS was accounted for by including information on previous calves (survival of the previous calf, total number of previous calves recorded for each female, the number of known calves surviving to year 1–2, or the number not surviving to year 1–2). In addition, we investigated the effect of calendar year and allowed for variation between individual females by including female ID as a random factor. Effects of IBI, numbers of previous calves and year were fitted as smoothers. The complexity of the fitted smoothers was limited using k = 4, due to most explanatory variables having too few unique values to allow an unconstrained smoother to be fitted. Since the causal relationship between IBI and calf birth could operate in both directions, a further GAMM was performed, with IBI (in months) as the (Poisson) response variable. The explanatory variables used were the same as for the probability of birth model (excluding IBI).

### Calving rates and annual fecundity

The annual number of births was obtained from counts of the individual new-born calves recorded during each year. Estimates of calving rates were calculated by dividing the annual number of new-borns by the number of animals using the study area each year, obtained from individual counts of identifiable dolphins. Calving rates for known individuals were calculated as the number of known calves produced by the female as a proportion of the years in which she was encountered since becoming reproductively-active (taken as the year before the production of the first known calf, given that the period of pregnancy is approximately one year (e.g. [18]).

Longitudinal photo-identification data were used to verify the minimum number of sexually mature females seen in the study area each year (including mature females that were no longer reproductively-active), from which annual estimates of fecundity could then be determined (after Fruet *et al*. [19]):

$${\hat{F}}_{i}=\frac{1}{2n}\times \sum_{i=1}^{n}\frac{N_{ci}}{N_{mi}}$$

where ${\hat{F}}_{i}$ is the estimated fecundity in year *i*; *n* is the total number of years sighted; N_ci_ is the number of calves born in year *i*; and *N*_*mi*_ is the number of mature females identified in year *i*.

### Annual and seasonal trends in calf births and calf survivability

Throughout the study, the first and last sightings for all known calves were recorded. Using the criteria proposed by Henderson *et al*. [20], birth months were subsequently assigned if one of the following conditions was met: (i) the mother was seen without a calf in the month prior to the first sighting of the calf; (ii) the mother was seen without a calf in the month of the first sighting of the calf; or (iii) the photos of the calf suggested it was a neonate (small size, indented foetal folds and/or floppy dorsal fin).

The seasonality of births and the peak birth period were examined for all assigned calves in the population. The pooled number of births per month was then plotted against sea temperature measurements recorded *in situ* during encounters from a calibrated temperature sensor within a transom-mounted transducer (Raymarine Inc. UK). Calf survival (by birth month) was also examined using GAMMs, to assess whether new-borns were more likely to perish if born late in the breeding season.

All GAMMs were fitted using R 3.1.2 [21] and Brodgar 2.7.4 (Highland Statistics Ltd., UK). Models were initially fitted for single explanatory variables, followed by forwards and backwards selection to find the best combination of explanatory variables, based on Akaike information criterion (AIC) values (where the model with the lowest AIC value was deemed to be the most parsimonious). Model outputs were examined for patterns in residuals and the existence of influential data points.

## Results

Between May and October 1997 to 2016, 530 encounters with bottlenose dolphins were recorded in the southern outer MF study area over 415 survey days. Group sizes varied between 2 and 70 animals, with a mean of 14.2. From a total of 102 females identified during the study period, 74 were seen to be reproductively active during the study period, producing at least one or more known calves. Approximately 33% of the reproductively active females (*n* = 23) produced just one calf during the study period, of which eleven females were known, first-time mothers. Thirty-two females produced three or more calves during the 20 years, and the maximum number of calves produced by any known female was seven (S1 Appendix).

### Calving rates, fecundity and the seasonality of births

**Fig 3:**
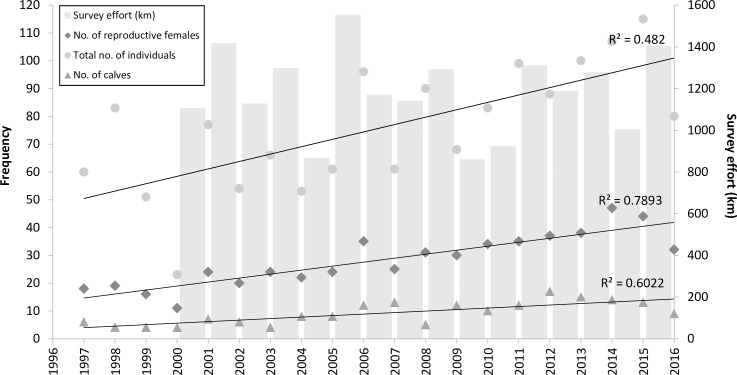
The total number of individuals, number of reproductive female bottlenose dolphins and the number of calves recorded in the outer southern Moray Firth study area from 1997 to 2016 inclusive.

A total of 193 new-borns were recorded during the study period. The annual number of new-borns ranged from 4 to 17 per year (mean ± SE = 9 ± 4; median = 7) (Table 1) with the number of annual births showing a general increase during the study period, coincident with an increasing number of reproductively-active females and increasing number of individuals recorded in the study area respectively (Fig 3). These patterns were not attributable to survey effort, as there was no significant correlation between annual survey effort and the number of individuals (*r* = -0.11, *t* = -0.41, *P* = 0.69), adult females (*r* = -0.05, *t* = -0.18, *P* = 0.86) or newborn calves (*r* = 0.15, *t* = 0.6, *P* = 0.56) recorded. Annual calving rates for the population ranged from 0.05 to 0.21 with a mean of 0.12 ± 0.05 (12%), whilst the annual fecundity in females ranged from 0.08 to 0.23 with a mean of 0.16 ± 0.04 (16%) (Table 1). Individual calving rates also ranged widely between reproductive females, from 0.14 to 0.57.

**Table 1:** The reproductive parameters of female bottlenose dolphins estimated from long-term mark-recapture studies in the southern outer Moray Firth from 1997 to 2016 inclusive. Weaning age for survival was considered to be to two years of age, after which calves were regarded to be fully independent of their mothers. See text for information on how RS, calving rate and fecundity were calculated.

STUDY YEAR	1997	1998	1999	2000	2001	2002	2003	2004	2005	2006	2007	2008	2009	2010	2011	2012	2013	2014	2015	2016	Mean (SE)
Method *	^F^	^F^	^F^	^F^	^F^	^F^	^F^	^F^	^F^	^F^	^D^	^D^	^D^	^D^	^D^	^D^	^D^	^D^	^D^	^D^	
Survey effort (km)	-	-	-	-	1108	1418	1129	1299	869	1556	1172	1142	1293	861	924	1311	1189	1278	1004	1405	1184 (201)
No. of individuals captured	60	83	51	23	77	54	66	53	61	96	61	90	68	83	99	88	99	107	115	80	76 (23)
No. adult females recorded	23	22	16	11	19	19	22	23	25	32	18	31	25	30	32	36	37	36	39	30	28 (10)
No. newborn calves	6	4	4	4	7	6	4	8	7	13	13	5	12	10	12	17	15	14	13	9	9 (4)
No. calves surviving to weaning	5	4	2	4	7	5	4	6	7	13	12	5	10	9	12	12	10	12	11	n/a	8 (3)
Reproductive success (RS)	0.67	1.00	0.50	1.00	1.00	0.83	1.00	0.75	1.00	1.00	0.92	1.00	0.83	0.90	0.83	0.76	0.67	0.86	0.85	n/a	0.86 (0.14)
Crude calving rate	0.12	0.05	0.08	0.17	0.09	0.11	0.06	0.17	0.12	0.14	0.21	0.06	0.18	0.12	0.12	0.19	0.15	0.13	0.11	0.11	0.12 (0.05)
Fecundity	0.17	0.11	0.13	0.18	0.15	0.15	0.08	0.18	0.17	0.17	0.26	0.08	0.20	0.15	0.17	0.23	0.20	0.15	0.15	0.14	0.16 (0.04)

* F = 35mm film, D = digital photography

**Fig 4:**
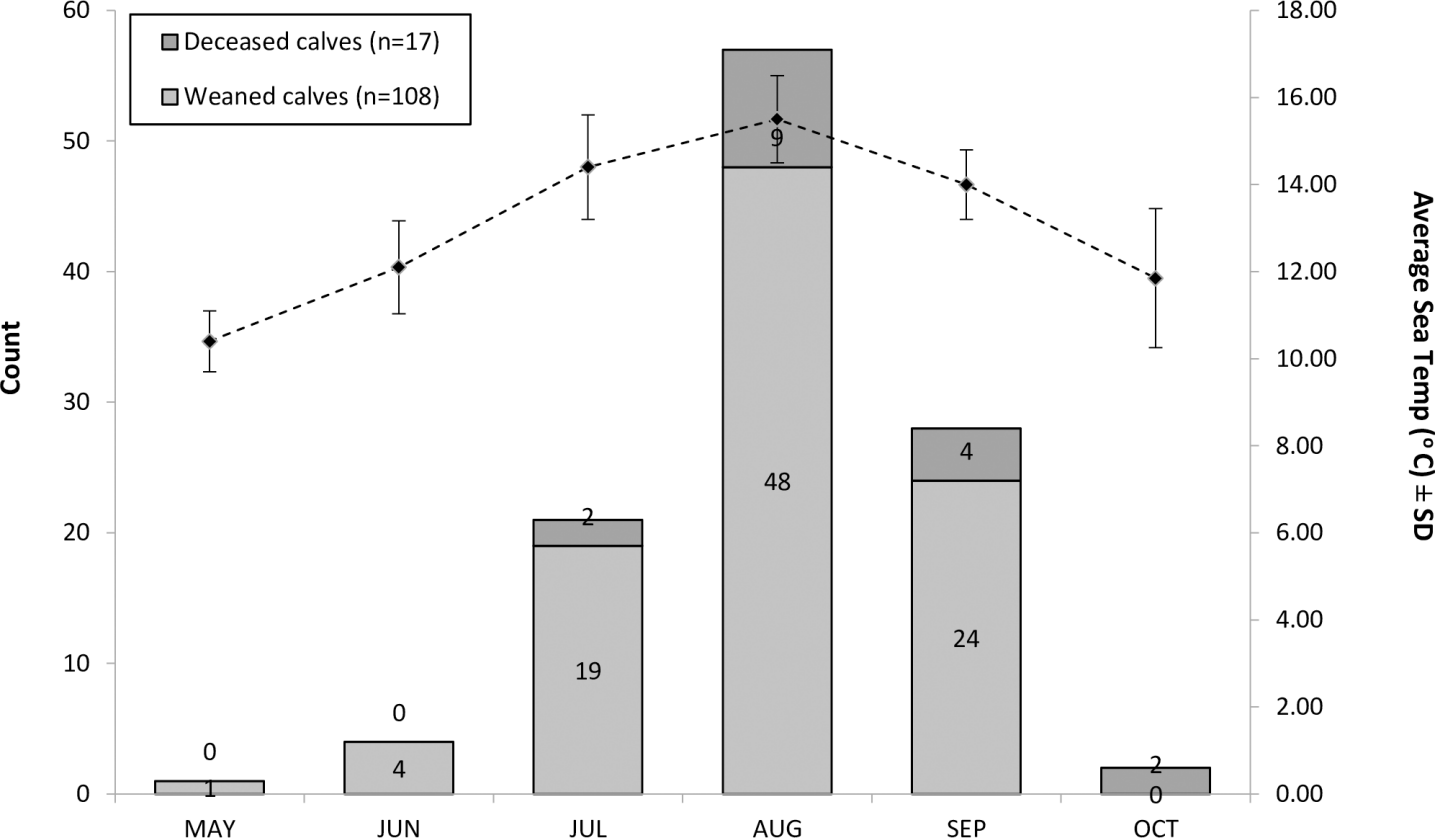
The seasonality of bottlenose dolphin births in the outer southern Moray Firth and the average monthly sea temperatures measured during encounters.

**Fig 5:**
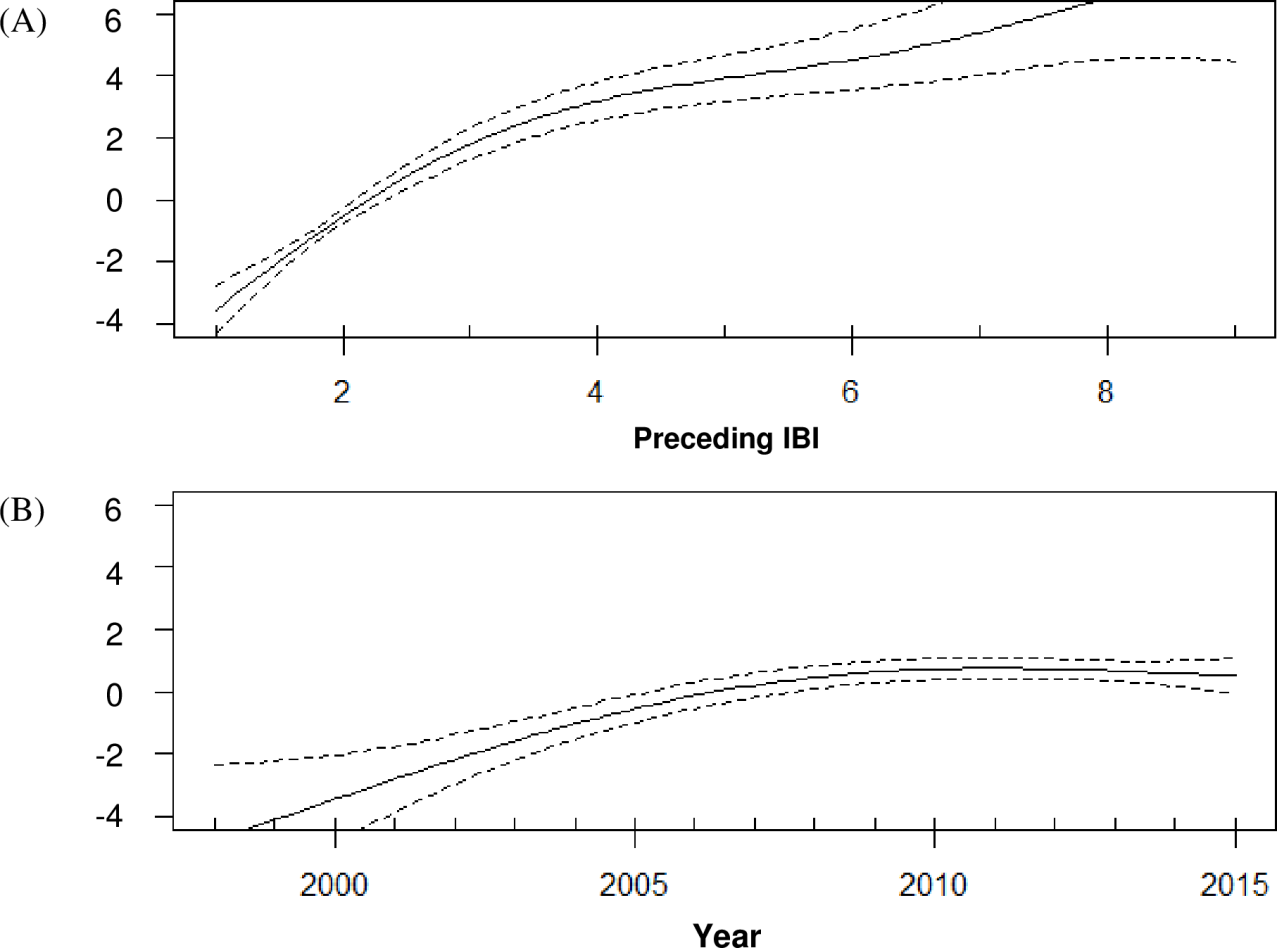
GAMM results showing smoothers for effects of: (A) time since previous birth in years and (B) the calendar year, on the likelihood of calf births. The strength and direction of the effect is shown by the y-axis. The dotted lines correspond to 95% confidence intervals.

Month of birth was successfully assigned for 126 calves from 57 known mothers. New-born calves were produced during all months of the study period (May to October inclusive), but the majority (94%) were born in July to September, with a peak in births (46%, *n* = 57) during August, coinciding with the annual peak in regional sea temperatures (Fig 4). The binomial GAMM investigating whether a female gave birth in a given year, included the IBI (*P* <0.0001, showing a positive effect up to 3 years since the last birth) (Fig 5A), survival of the previous calf (to year one) (*P* <0.0001, negative effect) and the calendar year (*P* <0.0001, positive effect) (Fig 5B).

### Inter-birth intervals (IBI)

**Fig 6:**
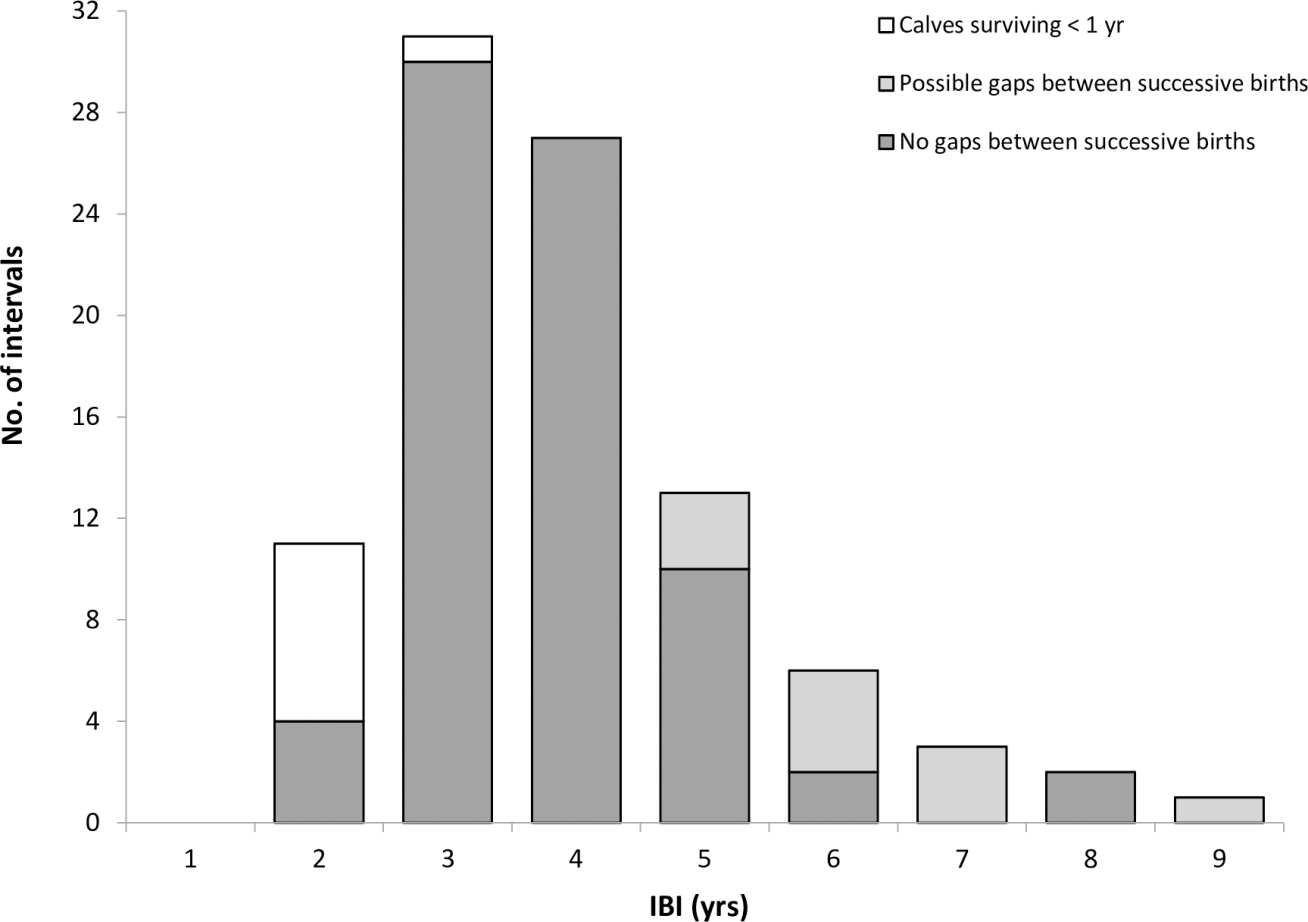
The range of IBIs documented for female *T*. *truncatus* in the outer southern Moray Firth study area, relative to previous calving success and possible gaps in reproductive histories.

Using the first approach for IBI determination, 110 IBIs were determined from 50 reproductive females, with IBIs ranging from 2 to 9 years with a mean (± SE) of 3.80 ± 1.40 years and mode of 4 (Fig 6). Using the second approach, where long IBIs (>5 years) were excluded, similar results were obtained (mean IBI = 3.56 ± 1.19 years, mode = 4 (*n* = 96)).

IBIs were significantly lower in females experiencing a calf loss (mean 2.02 ± 0.26 years; *t* = 10.86, *P* <0.05), with 87% of these females giving birth within two years following the loss of their calf. Conversely, observed IBIs between first and second-born surviving calves were seen to be much longer in duration (mean = 4.84 ± 1.27 years; *t* = -3.22, *P* = 0.004) than all subsequent IBIs in multiparous females. Indeed, the results from the GAMMs confirmed that the IBI preceding a calf birth was shorter if the previous calf did not survive to year 1 (*P* = 0.007) and the IBIs also decreased as the number of calves the female produced increased (*P* <0.0001). In known older females (*n* = 10), extended IBIs of 6 to 9 years were commonly observed with approaching reproductive senescence.

### Calf survival and reproductive success (RS)

The fate of 141 calves born to 54 females was tracked during the study period, of which 83% (*n* = 116) were raised to year 2–3. Approximately 46% of all detected calf mortalities were of calves of primiparous females, with only 13 of the 24 primiparous females identified in this study raising their first-born calf to weaning.

Seventeen calves with a known birth month died pre-weaning (year 0–1), of which 88% were born between August and October (Fig 4). A further five calf mortalities were recorded in year 1–2. Calf survival was lower in females who had previous calves that had died, which was the only explanatory variable found to have a significant effect on calf survival (*P* <0.0001).

**Fig 7:**
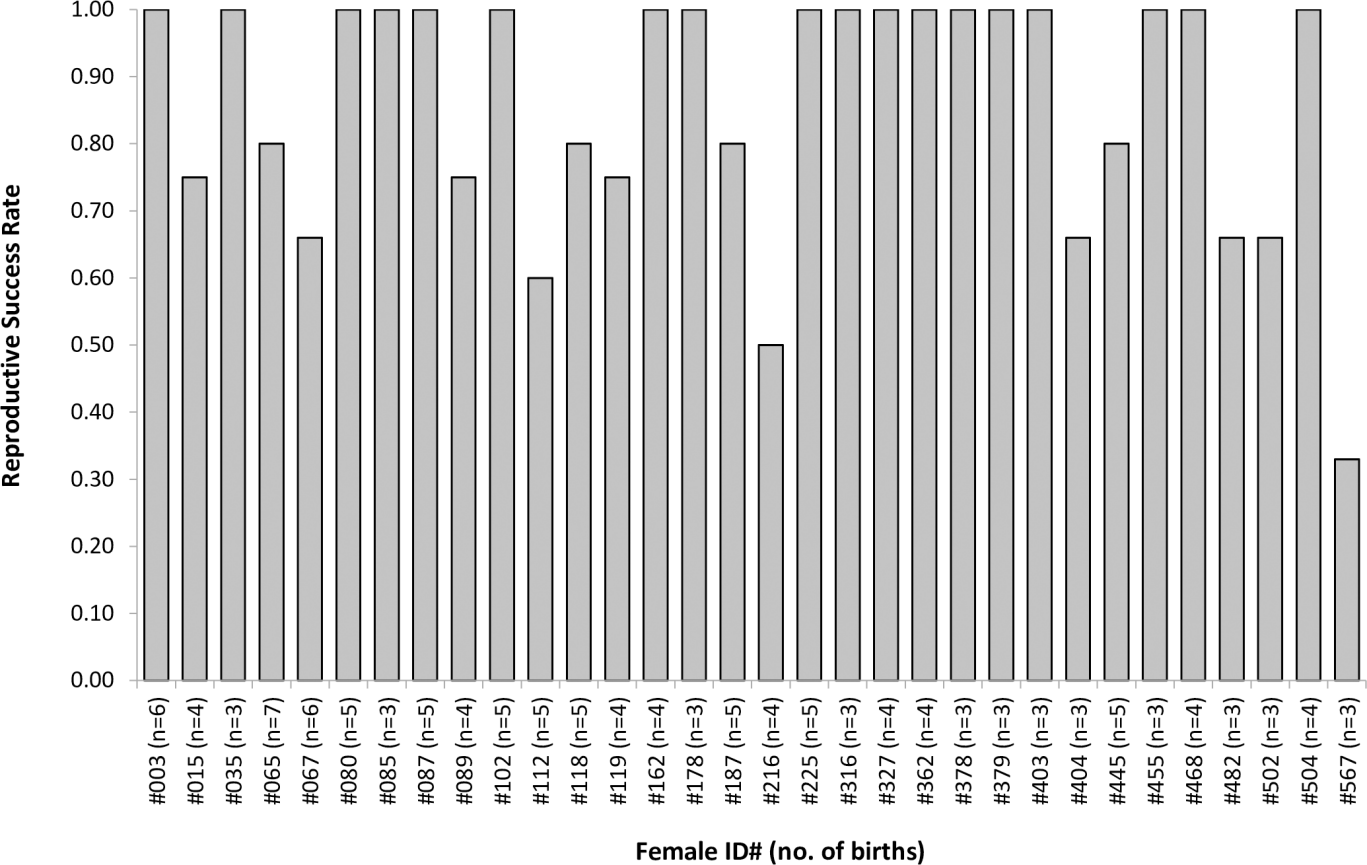
Individual variation in reproductive success observed in female bottlenose dolphins from the Moray Firth study population for mothers with ≥3 documented births of known fate. Unless already deceased, any calves born towards the end of the sampling period (in 2016) were not included, as they had not reached the minimum weaning age.

Overall RS in this population varied from one year to the next, ranging from 0.50 to 1.0 (50 to 100% success) with a mean of 0.86 ± 0.14 (Table 1). Individual RS was also highly variable between multiparous females producing three or more calves (*n* = 32), of which 18 successfully raised all their offspring to weaning (*n* = 70 calves), whilst one mother (ID#567) only managed to raise one out of three known calves during the study period (Fig 7).

### Age of females at first calf production

Sixteen of the reproductive females were tracked from birth. Eleven of these produced their first known calves at ages 8 and 9, but two primiparous females gave birth at age 6, suggesting that sexual maturity was attained as early as 5 years of age. One female known from birth produced her first known calf at age 13, although earlier calving attempts may have been undetected. Excluding this individual, the estimated age at first calf production in this population was between 6 and 10 years of age with a mean of 8.2 ± 1.1.

## Discussion

Female bottlenose dolphins attained sexual maturity at a young age in this North Sea population—from as early as five years with a minimum age of six at first reproduction. The estimated IBI of 3.8 years was comparable to that recorded in other *Tursiops* populations (e.g. [20, 22–25]), although Fruet *et al*. [19] reported shorter IBIs (3.3 years, mode of 2) for animals from the southwest Atlantic. Low IBIs between 2 and 2.25 years were also observed in three females with surviving calves in the present study. One female (ID#065), for example, produced four successive calves in a row with just a two-year interval between each birth. In primate species, lowered IBIs are reported for females receiving supplementary care for their offspring from other group members (e.g. [26–27]) which may also be relevant in group-living cetaceans (e.g. [28]). In the present study, however, the lowest IBIs were typically observed in females experiencing early calf loss. Conversely, the longest intervals were observed in ageing females presumed to be approaching reproductive senescence. In addition, significantly higher IBIs were observed between surviving first and second-born calves in young mothers, presumably reflecting the high energetic cost incurred in first calf production. Inevitably, a trade-off must exist between growth and reproduction in young females [29], and as such the observed intervals provided a comparative measure of the individual cost of reproduction and the level of investment made between females.

At 83%, the survival rate of calves in the present study was similar to that observed in other long-term bottlenose dolphin studies (e.g. 81% in Sarasota Bay, USA [30]; 86% in Doubtful Sound, New Zealand [31]). Of all known mortalities, however, more than 45% were attributed to primiparous females in the present study. In birth pulse communities (e.g. [19–20, 32]), the timing of births is thought to be crucially important for the survival of calves. However, a recent study by Cheney *et al*. [33] concluded that calf length, rather than birth month, was the best predictor of first-year survival in the MF study population. Smaller females invariably produce smaller offspring (e.g. [34]) which might have implications for thermoregulatory-related stress in undersized calves. On the other hand, larger females probably have greater resources to invest in their young during embryonic and/or post-natal development (e.g. [35]) or may simply be better at foraging. According to Krützen *et al*. (2004) [36], young females are also more susceptible to paternal inbreeding, which may result in reduced genetic fitness [37], whilst the calves of less experienced mothers are potentially most vulnerable to infanticidal attack [38]. A disproportionately high maternal transfer of accumulated polychlorinated hydrocarbons (PCBs) is also noted in primiparous females [39–40], with links having been documented between perinatal PCB exposure and brain cell damage [41], thyroid inhibition [42] and immunosuppression [43], which might further account for the low survival rate of first-born calves recorded in the present study.

Whilst both age and breeding experience in female dolphins may be important for offspring survival (e.g. [44]), reproductive output is also age-specific, such that the rate of calving in older females identified in the present study showed a progressive decline over time. Fruet *et al*. [19] proposed that maturing females changed their role from that of “breeding” to more predominantly “nursing” individuals with ageing, which might effectively serve to increase calf survival and achieve greater long-term population viability. Kinship may be important in such allomaternal behaviour (e.g. [45–46]), but evidence increasingly suggests that helpers may be entirely unrelated and cooperation is maintained by mutual reciprocity instead [47–48]. In pregnant dolphins and in mothers with young calves, however, reproductive state is thought to be the most influential determinant in the formation of preferred associations [48]. In the MF, associations between animals of a similar age and reproductive state are commonly observed (e.g. [49–50]), with bonds having been established since infancy, such that known female associates include the daughters of their mother’s closest associates with whom they grew up with or spent time with as juveniles.

Group living is evidently beneficial for collective reproductive fitness in social mammals [46,51], and the formation of bonds by MF females may afford enhanced access to resources, mutual protection from sexually-coercive males, increased group vigilance and other social benefits necessary for calving [7,15,52]. Comparative studies of several mammalian groups have shown that larger group sizes inevitably produce larger numbers of surviving offspring (e.g. [53,54]). However, ranking and dominance may also be significant for RS in social delphinids, by helping females to establish and maintain access to optimal foraging areas, for example (e.g. [15]). In chimpanzee (*Pan troglodytes*) societies, high-ranking females display higher rates of offspring survival and more rapid production of young than lower-ranking females [55]. In addition, daughters of low-ranking females may mature as much as four years later than those of high-ranking females [56]. The age of sexual maturation in the 16 primiparous females identified in the present study varied widely, from 5 to 12 years, which might be explained by the respective dominance ranking of their mothers. According to Samuels and Gifford [57], dominance is age-ordered and stable amongst cooperating female *T*. *truncatus*. Thus, mother identity could be essential for calf survival in the MF, such that *which* females give birth and *when* may be pivotal to the viability of this small, vulnerable resident population [20].

In conclusion, intra-specific variability in female RS in the MF could be attributed to many factors, including maternal age, size and breeding experience, dominance and parity of the mother, the survival of previous calves, individual associations and group size, social factors, and resource availability. Clearly not all individuals within a population provide the same value or function to its structure (e.g. [58–59]), and in the present study the individual heterogeneity in female RS could be attributed to certain females being more successful, and consequently more important, for the viability of the population than others. A small decrease in this population could subsequently have an unpredictable effect on overall reproductive output if these important breeders are lost. Thus, the present findings might be highly significant to management, as the identification of demographic groups containing the most reproductively successful females, i.e., “good” mothers, might be highly desirable to conservation efforts for their protection. One precautionary approach, for example, would be to regularly assess which females are present, where and when, thereby allowing evaluations of potential pressures (i.e. disturbance by wildlife tour boat operators) negatively impacting these animals during calving or nursing phases.

Whilst the detrimental effects that demographic stochasticity plays in the dynamics of small populations are well known (e.g. [60]), the mechanisms influencing variations at an individual level are still poorly understood [61]. However, the present study suggests that RS in small populations may invariably rely on just a small number of individuals, which dramatically increases the susceptibility of small populations to environmental change and/or harmful anthropogenic impacts. Continued monitoring of the MF population remains necessary to provide robust estimates of life-history parameters, e.g. fecundity rates recently published by Arso Civil *et al*. [62], which will not only improve our ability to revise and advance the conservation of this potentially vulnerable population, but will also serve to further our ability to inform regulators of the potential impacts of ongoing developments (i.e., offshore windfarm installations, oil and gas activities) presently affecting these animals in northeast Scottish coastal waters. More broadly, where regulators are under increasing pressure to ensure adequate protection for protected species, such as the bottlenose dolphin, at a time when a growing number of cumulative detrimental impacts are imposed on the marine environment [63–64], the need for appropriate long-term monitoring of long-lived species with expansive home ranges becomes increasingly evident.

## Supporting information

S1 Appendix Sightings and calving histories of reproductively active females listed by their unique identification number (ID#) as recorded by CRRU in the southern Moray Firth study area from 1997 to 2016 inclusive. (DOCX)
