# Supplementary material for: Female reproductive success and calf survival in a North Sea coastal bottlenose dolphin (Tursiops truncatus) population
Source: PLoS One. 2017 Sep 20;12(9):e0185000. doi: 10.1371/journal.pone.0185000 (PMC5607131; doi:10.1371/journal.pone.0185000)
Supplement: S1 Appendix — (DOCX) [file pone.0185000.s001.docx]

**Appendix S1**

Sightings and calving histories of reproductively active females listed by their unique identification number (ID#) recorded in the southern Moray Firth study area from 1997 to 2016 inclusive: “0” = not sighted, “1” = sighted; “●” = sighted with newborn calf; “◙” = sighted with first-born calf (where the mother was successfully tracked from birth by CRRU or the University of Aberdeen Lighthouse Field Station (Barbara Cheney, pers. comm.) respectively); “●^1^” = estimated year of birth for calves born prior to 1997 (when the photo-identification study commenced); “^†^” = confirmed calf mortality; and “-” = sightings from the year of birth prior to sexual maturation.

| **ID#** | **No. of calves** | **1995** | **1996** | **1997** | **1998** | **1999** | **2000** | **2001** | **2002** | **2003** | **2004** | **2005** | **2006** | **2007** | **2008** | **2009** | **2010** | **2011** | **2012** | **2013** | **2014** | **2015** | **2016** | **No. of sightings** |
| --- | --- | --- | --- | --- | --- | --- | --- | --- | --- | --- | --- | --- | --- | --- | --- | --- | --- | --- | --- | --- | --- | --- | --- | --- |
| 003 | 6 |  |  | ● | 1 | 0 | 0 | 1 | ● | 1 | 1 | 1 | ● | 1 | 1 | ● | 1 | 1 | 1 | ● | 1 | 1 | ● | 138 |
| 015 | 4 | ●^1^ |  | 1 | 1 | 0 | 0 | 1 | 0 | 1 | ● | 1 | 0 | ●^†^ | 1 | 1 | ● | 0 | 0 | 0 | 0 | 0 | 0 | 28 |
| 026 | 2 |  |  | 1 | 1 | 1 | ● | 1 | 0 | 0 | 0 | 0 | ● | 0 | 0 | 0 | 0 | 0 | 0 | 0 | 0 | 0 | 0 | 11 |
| 032 | 1 |  |  | 1 | 0 | 1 | 0 | 0 | 0 | 0 | 0 | 0 | 1 | 0 | 0 | 0 | ● | 0 | 0 | 0 | 1 | 1 | 0 | 9 |
| 035 | 3 |  |  | 0 | 0 | 0 | 0 | 1 | ● | 1 | 1 | 1 | 1 | 1 | ● | 1 | 1 | 1 | 1 | ● | 1 | 1 | 1 | 121 |
| 046 | 1 |  |  | 1 | 1 | 1 | ● | 0 | 0 | 1 | 0 | 0 | 0 | 0 | 0 | 0 | 0 | 0 | 0 | 0 | 0 | 0 | 0 | 10 |
| 055 | 2 |  |  | 1 | 1 | 1 | 0 | 0 | 0 | 0 | ● | 0 | 0 | 0 | 0 | 0 | 0 | 0 | 0 | 0 | 1 | ● | 0 | 11 |
| 065 | 7 | - | - | ◙^†^ | 1 | 0 | 0 | 1 | ● | 1 | 1 | 1 | 1 | ● | 1 | 1 | ● | 1 | ● | 1 | ● | 1 | ● | 130 |
| 067 | 6 |  | ●^1^ | 1 | 1 | 1 | 0 | 0 | ● | 1 | 1 | 1 | ● | 1 | 1 | ● | 1 | 1 | 1 | ●^†^ | 1 | ● | 1 | 165 |
| 072 | 1 |  |  | ● | 1 | 0 | 0 | 0 | 0 | 0 | 0 | 0 | 0 | 0 | 0 | 0 | 0 | 0 | 0 | 0 | 0 | 0 | 0 | 12 |
| 078 | 2 |  |  | ● | 1 | 1 | 1 | 0 | 0 | 0 | 0 | 0 | ● | 0 | 0 | 0 | 0 | 0 | 0 | 0 | 0 | 0 | 0 | 8 |
| 080 | 3 |  |  | 1 | 0 | 1 | 0 | 1 | ● | 0 | 1 | 0 | 1 | ● | 1 | 0 | 0 | ● | 0 | 0 | 0 | 1 | 0 | 16 |
| 081 | 2 |  |  | ● | 1 | 0 | 0 | ● | 1 | 1 | 1 | 0 | 0 | 0 | 0 | 0 | 0 | 0 | 0 | 0 | 1 | 0 | 0 | 16 |
| 085 | 3 |  |  | 0 | 0 | 0 | 0 | 0 | 0 | 0 | 0 | 0 | 1 | ● | 1 | 1 | 1 | ● | 1 | 1 | ● | 1 | 1 | 22 |
| 087 | 5 |  |  | 1 | 1 | 0 | 0 | ● | 1 | 1 | 0 | 0 | ● | 0 | 0 | ● | 1 | 1 | ●^†^ | 1 | ● | 0 | 1 | 15 |
| 089 | 4 |  |  | ● | 1 | 0 | 0 | 1 | 1 | ● | 0 | 1 | 1 | ● | 1 | 0 | 0 | 1 | ● | 1 | 1 | 0 | 0 | 19 |
| **ID #** | **No. of calves** | **1995** | **1996** | **1997** | **1998** | **1999** | **2000** | **2001** | **2002** | **2003** | **2004** | **2005** | **2006** | **2007** | **2008** | **2009** | **2010** | **2011** | **2012** | **2013** | **2014** | **2015** | **2016** | **No. of sightings** |
| 102 | 5 | ●^1^ |  | 1 | 1 | 1 | 1 | ● | 1 | 0 | 0 | 0 | ● | 0 | 0 | 0 | ● | 0 | 0 | ● | 1 | 0 | 0 | 14 |
| 112 | 5 |  |  | 0 | 0 | 0 | 0 | ● | 0 | 0 | ● | 0 | 1 | ● | 1 | 1 | ● | 1 | ● | 1 | 1 | 1 | 0 | 25 |
| 118 | 5 |  |  | - | - | 1 | ◙ | 1 | 1 | 1 | 1 | ● | 1 | 1 | ● | 1 | 1 | ●^†^ | 1 | ● | 1 | 1 | 1 | 120 |
| 119 | 4 |  |  | 0 | 0 | 0 | 0 | 0 | 1 | ● | 1 | 1 | ● | 1 | 1 | 1 | ●^†^ | 1 | ● | 1 | 1 | 1 | 1 | 128 |
| 138 | 2 |  |  | 1 | ● | 0 | 0 | 0 | 0 | 0 | ● | 1 | 0 | 0 | 0 | 0 | 0 | 0 | 0 | 0 | 0 | 0 | 0 | 8 |
| 162 | 4 |  |  | 0 | 0 | 1 | 1 | ● | 0 | 1 | 1 | ● | 1 | 0 | 0 | 1 | 1 | ● | 1 | 1 | 1 | ● | 1 | 61 |
| 178 | 3 |  |  | 1 | 0 | 1 | 1 | 0 | 0 | 0 | 0 | 1 | ● | 0 | 0 | 0 | ● | 0 | 0 | ● | 1 | 1 | 0 | 12 |
| 187 | 5 |  |  | - | - | ● | 1 | 1 | 0 | 1 | ●^†^ | 1 | ● | 1 | 1 | ● | 1 | 1 | ● | 1 | 1 | 0 | 0 | 129 |
| 191 | 1 |  |  | 0 | 1 | ● | 1 | 1 | 1 | 0 | 0 | 0 | 0 | 0 | 0 | 0 | 0 | 0 | 0 | 0 | 0 | 0 | 0 | 10 |
| 216 | 4 |  |  | 0 | 0 | 1 | 1 | 1 | ●^†^ | 1 | 1 | 1 | 1 | ●^†^ | 1 | 1 | 1 | ● | 1 | 1 | ● | 1 | 1 | 99 |
| 225 | 5 |  |  | 0 | 0 | 0 | ● | 1 | 1 | 1 | ● | 1 | 1 | ● | 1 | 1 | ● | 1 | 1 | 1 | ● | 1 | 1 | 104 |
| 252 | 1 |  |  | 0 | 0 | 0 | 0 | 1 | 1 | 1 | 0 | 0 | 0 | 0 | 1 | 1 | ● | 1 | 0 | 0 | 0 | 0 | ● | 9 |
| 253 | 2 |  |  |  |  |  | - | - | - | - | - | - | - | - | - | - | - | - | - | ◙^†^ | 1 | ● | 1 | 49 |
| 302 | 1 |  |  | 0 | 0 | 0 | 0 | 0 | 1 | 1 | 0 | 1 | ●^†^ | 1 | 1 | 0 | 0 | 0 | 0 | 0 | 0 | 0 | 0 | 11 |
| 316 | 3 | - | - | - | - | - | - | - | 1 | ◙ | 0 | 0 | 1 | 0 | 1 | 1 | ● | 0 | 1 | 0 | 0 | ● | 0 | 9 |
| 319 | 3 | - | - | - | - | - | - | - | - | - | - | - | 0 | ◙ | 0 | 0 | 0 | 0 | 0 | 0 | ● | 1 | ● | 7 |
| 327 | 4 |  |  | 0 | 0 | 0 | 0 | 0 | 0 | 0 | 1 | 1 | ● | 1 | 1 | ● | 1 | ● | 1 | 1 | ● | 1 | 1 | 75 |
| 359 | 1 |  |  |  |  |  |  |  |  |  | - | - | - | - | - | - | - | - | 1 | ◙^†^ | 1 | 1 | 0 | 57 |
| 362 | 4 | ◙^1^ |  | 0 | 1 | 0 | 0 | ● | 1 | 1 | 1 | ● | 1 | 0 | 1 | 0 | 0 | 0 | ● | 0 | 0 | 0 | 1 | 23 |
| 374 | 1 |  |  | 1 | 1 | 0 | 0 | 0 | 0 | 0 | 0 | 0 | ● | 1 | 1 | 0 | 0 | 0 | 0 | 0 | 0 | 0 | 0 | 7 |
| 378 | 3 |  |  | 0 | 0 | 0 | 0 | 0 | 0 | 0 | 1 | ● | 1 | 1 | 1 | ● | 1 | 1 | ● | 1 | 1 | 1 | 0 | 30 |
| 379 | 3 |  |  | 0 | 0 | 0 | 0 | 0 | 0 | 0 | 0 | 1 | 1 | ● | 1 | 0 | 0 | ● | 0 | 0 | ● | 0 | 1 | 21 |
| 385 | 1 |  |  |  |  |  |  |  |  |  |  |  | - | - | - | - | - | - | 1 | ◙ | 1 | 0 | 0 | 53 |
| 387 | 2 |  |  |  |  |  |  |  | - | - | - | - | - | - | 1 | ◙^†^ | 1 | ● | 0 | 0 | 0 | 0 | 0 | 10 |
| 389 | 2 |  |  | 0 | 1 | 1 | 0 | 1 | 0 | 1 | ●^†^ | 1 | 1 | 1 | 1 | 0 | 0 | 1 | 1 | ● | 1 | 1 | 0 | 54 |
| **ID #** | **No. of calves** | **1995** | **1996** | **1997** | **1998** | **1999** | **2000** | **2001** | **2002** | **2003** | **2004** | **2005** | **2006** | **2007** | **2008** | **2009** | **2010** | **2011** | **2012** | **2013** | **2014** | **2015** | **2016** | **No. of sightings** |
| 396 | 2 |  |  |  |  |  |  | - | - | - | - | - | - | - | - | 1 | ◙ | 1 | 1 | ● | 1 | 1 | 1 | 31 |
| 403 | 3 |  |  | 0 | 0 | 0 | 0 | 1 | 0 | 0 | 0 | ● | 1 | 0 | 0 | ● | 0 | 0 | ● | 1 | 0 | 0 | 0 | 16 |
| 404 | 3 |  |  | 0 | 0 | 0 | 0 | 0 | 0 | 0 | 0 | 0 | 1 | 0 | 0 | ● | 1 | 1 | ●^†^ | 0 | ● | 1 | 1 | 24 |
| 410 | 2 |  |  |  | - | - | - | - | - | - | - | - | - | - | - | - | 1 | ◙^†^ | 0 | ● | 0 | 0 | 0 | 8 |
| 432 | 3 |  |  | 0 | 0 | 0 | 0 | 0 | 0 | 0 | 0 | ● | 1 | 1 | ● | 1 | 1 | 1 | 1 | 1 | ● | 1 | 1 | 89 |
| 436 | 1 |  |  |  |  |  |  |  |  |  |  |  |  | - | - | - | - | - | - | - | 1 | ◙ | 1 | 83 |
| 445 | 5 | - | - | - | - | - | - | - | 1 | ◙ | 0 | 0 | ● | 0 | 1 | ● | 1 | 0 | ●^†^ | 0 | ● | 1 | 1 | 15 |
| 453 | 1 |  |  |  |  |  |  |  |  |  |  |  |  | - | - | - | - | - | - | - | 1 | ◙ | 0 | 9 |
| 455 | 3 |  |  | - | - | - | - | 1 | ● | 1 | ◙ | 1 | 1 | ● | 1 | 0 | 0 | 0 | 0 | 0 | 0 | 0 | 0 | 16 |
| 463 | 2 |  |  | 0 | 0 | 0 | 0 | 0 | 0 | 0 | 0 | 0 | 0 | 1 | ● | 0 | 0 | 1 | ● | 0 | 0 | 0 | 0 | 5 |
| 465 | 1 |  |  |  |  |  |  |  |  |  | - | - | - | - | - | - | - | - | 1 | ◙ | 0 | 0 | 1 | 6 |
| 468 | 4 |  |  | 0 | ● | 1 | 0 | 0 | 0 | ● | 0 | 0 | 1 | ● | 1 | 0 | 0 | ● | 0 | 0 | 0 | 1 | 0 | 19 |
| 469 | 1 |  |  |  |  |  |  |  |  |  |  |  |  | - | - | - | - | - | - | - | - | 1 | ◙ | 6 |
| 478 | 1 |  |  |  |  |  |  |  |  |  |  |  |  |  |  | - | - | - | - | - | 1 | ◙ | 1 | 103 |
| 482 | 3 |  |  | 0 | 0 | 0 | 0 | 1 | 0 | 0 | 0 | ● | 1 | 0 | 1 | 1 | 1 | 1 | ●^†^ | 1 | ● | 1 | 0 | 83 |
| 486 | 2 |  |  |  |  |  |  |  |  |  | - | - | - | - | - | - | - | 1 | ◙^†^ | 1 | 1 | ● | 0 | 43 |
| 487 | 1 |  |  |  |  |  |  |  |  |  |  | - | - | - | - | - | - | - | - | 1 | ◙ | 1 | 0 | 42 |
| 498 | 2 |  |  |  |  |  |  |  |  |  |  | - | - | - | - | - | - | - | 1 | ◙ | 1 | 1 | ●^†^ | 70 |
| 502 | 3 |  |  | 0 | 0 | 0 | 0 | 0 | 0 | 0 | 0 | 0 | 0 | 0 | 0 | ● | 1 | 0 | 1 | ●^†^ | 1 | ● | 0 | 6 |
| 504 | 4 |  |  | 0 | 0 | 0 | 0 | 0 | 0 | 1 | ● | 0 | 0 | 0 | 0 | ● | 1 | 0 | ● | 0 | 1 | ● | 0 | 11 |
| 505 | 1 |  |  |  |  |  |  |  |  |  |  |  |  |  |  | - | - | - | - | - | - | 0 | ◙ | 3 |
| 506 | 2 |  |  |  |  |  |  |  |  | - | - | - | - | - | - | - | - | - | 1 | ◙ | 1 | 1 | ● | 126 |
| 511 | 2 |  |  | 0 | 0 | 0 | 0 | ● | 0 | 0 | 0 | 0 | 0 | 0 | 1 | ●^†^ | 1 | 1 | 0 | 1 | 1 | 1 | 1 | 16 |
| 517 | 1 |  |  | 0 | 0 | 0 | 0 | 0 | 0 | 0 | 0 | 0 | 0 | 0 | 0 | 0 | 1 | 0 | 0 | ● | 0 | 0 | 0 | 4 |
| 521 | 2 |  |  | 0 | 0 | 0 | 0 | 0 | 0 | 0 | 0 | 0 | 0 | 0 | 0 | 0 | 0 | 1 | ● | 1 | 1 | ● | 0 | 19 |
| **ID #** | **No. of calves** | **1995** | **1996** | **1997** | **1998** | **1999** | **2000** | **2001** | **2002** | **2003** | **2004** | **2005** | **2006** | **2007** | **2008** | **2009** | **2010** | **2011** | **2012** | **2013** | **2014** | **2015** | **2016** | **No. of sightings** |
| 526 | 2 |  |  | 0 | 0 | 0 | 0 | 0 | 0 | 0 | 0 | 0 | 0 | 0 | 0 | 0 | 1 | ◙ | 0 | 0 | 0 | ● | 0 | 4 |
| 529 | 1 |  |  | 0 | 0 | 0 | 0 | 0 | 0 | 0 | 0 | 0 | 0 | 0 | 0 | 0 | 0 | 1 | ◙ | 1 | 0 | 0 | 0 | 3 |
| 530 | 1 |  |  | 0 | 0 | 0 | 0 | 0 | 0 | 0 | 0 | 0 | 0 | 0 | 0 | 0 | 0 | 1 | ◙^†^ | 1 | 1 | 1 | 1 | 9 |
| 532 | 1 |  |  | 0 | 0 | 0 | 0 | 0 | 0 | 0 | 0 | 0 | 0 | 0 | 0 | 0 | 0 | 1 | 0 | ● | 1 | 0 | 0 | 4 |
| 567 | 3 |  |  |  |  |  |  | - | - | - | - | - | - | - | 0 | ◙ | 0 | 0 | 0 | ●^†^ | 1 | ● | 0 | 6 |
| 578 | 1 |  |  | 0 | 0 | 0 | 0 | 0 | 0 | 0 | 0 | 0 | 0 | 0 | 0 | ● | 0 | 0 | 0 | 1 | 0 | 0 | 0 | 3 |
| 634 | 1 |  |  |  |  |  |  |  |  |  | - | - | - | - | - | - | - | - | - | - | - | 0 | ◙ | 2 |
| 636 | 1 |  |  | 0 | 0 | 0 | 0 | 0 | 0 | 0 | 0 | 0 | 0 | 0 | 0 | 0 | ● | 0 | 0 | 0 | 0 | 1 | 1 | 4 |
